# Maternity ward management and COVID-19 pandemic: Experience of a single center in Northern Italy during lockdown

**DOI:** 10.18332/ejm/137605

**Published:** 2021-07-19

**Authors:** Nicola Cesano, Francesco D’Ambrosi, Giulia E. Cetera, Ilma F. Carbone, Matteo Di Maso, Manuela W. Ossola, Enrico Iurlaro, Enrico Ferrazzi

**Affiliations:** 1 Department of Woman, Child and Neonate, Fondazione IRCCS Ca’ Granda Ospedale Maggiore Policlinico, Milan, Italy; 2 Branch of Medical Statistics, Biometry and Epidemiology ‘G.A. Maccacaro’, Department of Clinical Sciences and Community Health, Faculty of Medicine, University of Milan, Milan, Italy; 3 Department of Clinical Sciences and Community Health, Faculty of Medicine, University of Milan, Milan, Italy; + Co-first authors

**Keywords:** COVID-19, delivery, maternal complication, neonatal complication, northern Italy, Sars-CoV-2 pandemic

## Abstract

**INTRODUCTION:**

The aim of our study is to describe the management of a maternity ward in a referral center during the COVID-19 pandemic and 2020 lockdown.

**METHODS:**

This is a retrospective single-center study. We analyzed the records of all women consecutively admitted to our delivery ward during lockdown and compared them with those of women admitted in the same period in 2019.

**RESULTS:**

The number of patients (1260) admitted to our department in 2020 was similar (1215) to that in 2019. Among patients admitted during lockdown, 50 presented with a Sars-CoV-2 infection (3.9%). In 2020, the number of antenatal check-ups was lower than in 2019 [7.9 (1.5) vs 8.2 (1.3), p<0.001] and the rate of labor inductions was higher [436 (34.6) vs 378 (31.1), p=0.008] although no difference in delivery mode was found. Moreover, women admitted during lockdown were more likely to give birth alone [140 (11.1) vs 50 (4.1), p<0.001]. However, during 2020, the rate of mother and newborn skinto-skin contact [1036 (82.2) vs 897 (73.8), p<0.001] and that of breastfeeding within 2 hours from birth [1003 (79.6) vs 830 (68.3), p<0.001] was higher. We found no significant differences in maternal or neonatal outcomes.

**CONCLUSIONS:**

Despite the COVID-19 pandemic, we were able to guarantee a safe birth assistance to all pregnant women, both for those infected and those not infected by Sars-CoV-2.

## INTRODUCTION

The COVID-19 pandemic is causing a profound impact on health services worldwide. In Northern Italy, one of the first areas in Europe to be hit by the pandemic^1-3^, the virus has spread significantly amongst the pregnant population, affecting antenatal care^4-9^.

The Italian government has taken extraordinary measures, to prevent the spread of the infection, including a national lockdown, which took place from 9 March to 18 May 2020. During the lockdown period, any movement for health reasons was always allowed^7^.

In order to manage the state of emergency, the National Health System relocated resources to reorganize human and logistical aid. Furthermore, the fear of contracting the infection in hospitals has reduced patients’ access to health services^4,7,8^.

The aim of our study is to describe the management of a maternity ward in a referral center during the COVID-19 pandemic and 2020 lockdown, and to investigate any differences in fetal and maternal outcomes between 2019 and 2020.

## METHODS

This retrospective transverse observational single-center study was carried out in our Institution Fondazione IRCCS Ca’ Granda, Ospedale Maggiore Policlinico, Milan, Italy, one of the six COVID-19 maternity hubs designed by the Regional Health Authority and the largest high-risk Maternity Unit in the metropolitan area of Milan, Lombardy^4,7^.

We provide a detailed description of the activity in our delivery ward which consists of 9 delivery rooms, 3 operating theatres, and 8 observation beds.

During the COVID-19 pandemic, we divided our delivery ward into two wings: one for patients tested positive for Sars-CoV-2 (3 delivery rooms and an operating theatre) and one for non-infected patients. Access to each area was independent, in order to prevent contact between positive and negative patients. Likewise, we divided the postpartum ward into two wings: one for women with COVID-19 (20 beds) and one for women tested negative (80 beds).

The delivery room staff consisted of 6 midwives, 4 gynecologists, 2 obstetrics and gynecology residents, 2 anesthetists, and 1 or 2 nurses per shift.

We retrospectively analyzed the records of all women consecutively admitted to our delivery room during lockdown from 1 March to 30 April 2020, and compared them with those admitted in the same period in 2019. All pregnant women admitted to our Institution to give birth were eligible for the present study.

At time of admission, all women were initially screened with a questionnaire for symptoms of upper respiratory tract infections (URTI) and contacts with people infected by Sars-CoV-2. All women who reported fever, respiratory symptoms or high-risk contacts were isolated in a dedicated area. All partners or relatives desiring to be present during birth were also screened. If screening resulted positive, they were not allowed to access the delivery room.

Only one family member per patient was allowed in and visits to the postpartum ward were not allowed.

The questionnaire was carried out by staff using individual protection devices such as eye protection, gowns, gloves and filtering face piece masks such as (FFP-2)^10^.

In accordance to the Italian National Guidelines, all women, both positive and negative for the screening questionnaire, were further screened by the means of a nasopharyngeal swab^11,12^. The swab was taken 48 hours before admission if this had been scheduled (e.g. induction to labor or elective caesarean section) or at time of admission if the patient needed urgent hospitalization (e.g. labor, premature rupture of membranes, vaginal bleeding).

Patients needing urgent care, who therefore could not wait for the result of the swab test to be admitted, or patients with a negative swab but a positive screening questionnaire, were treated as suspected cases of COVID-19.

All staff working in the wing of the delivery ward restricted to confirmed or suspected cases of COVID-19 used personal protective devices such as eye protection, gowns, gloves, and filtering face piece masks (FFP-2). Staff working in the area dedicated to women screened negative for COVID-19 wore glasses, gloves and surgical masks. All women and partners were required to wear a surgical mask regardless of their screening status.

We obtained data from the national CedAP database (Certificate of assistance at birth), which is filled in by health workers when a pregnant woman is admitted to the delivery ward.

The study was approved by the Institutional Review Board of Fondazione IRCCS Ca’ Granda, Ospedale Maggiore Policlinico, Milan, Italy (No.1512; date: April 2020).

### Statistical analysis

We performed a descriptive analysis of variables and continuous variables are reported in terms of mean and standard deviation (SD). Data were analyzed using the statistical package IBM SPSS 22.0 (New York, USA) and Excel for Windows 2010 (Microsoft Corp., Redmond, WA, USA).

## RESULTS

During the period considered in our study, 1260 women gave birth in our Institution, compared to 1215 patients who gave birth in the same time period in 2019.

Among the 1260 patients admitted to our delivery ward in the study period in 2020, 50 (3.9%) tested positive for Sars-CoV-2.

Maternal characteristics such as age, pregravidic BMI, citizenship, education level, and employment, were similar in the two groups (Table 1).

**Table 1:** Maternal and obstetric characteristics during the pandemic lockdown period compared to the same period in 2019

*Characteristics*	*2019 (n=1215) n (%)*	*2020 (n=1260) n (%)*	*p*
Italian citizen	920 (75.7)	933 (74.0)	0.34
Age (years), mean ± SD	34.8 ± 5.2	34.3 ± 5.5	0.21
**Education level**			0.21
Degree	710 (58.5)	688 (54.7)	
High school	386 (31.7)	425 (33.7)	
Middle school	111 (9.1)	139 (11.0)	
Primary school/none	8 (0.7)	8 (0.6)	
Unemployed	284 (23.4)	285 (22.6)	0.65
Multiparous	586 (48.2)	543 (43.1)	**0.01**
Number of visits during pregnancy, mean ± SD	8.2 ± 1.3	7.9 ± 1.5	**<0.001**
Ultrasound scan after 22 weeks	1180 (97.1)	1235 (98.0)	0.15
Gestational age at first check-up (weeks), mean ± SD	6.7 ± 1.6	6.9 ± 1.3	**0.002**
Number of fetal ultrasound scans	7.7 (2.0)	7.3 (1.9)	**<0.001**
Pregravidic BMI (kg/m^2^), mean ± SD	22.8 ± 4.1	22.5 ± 4.2	0.07
Pregnancy complications	197 (16.2)	196 (15.6)	0.65
IUGR	67 (5.5)	81 (6.4)	0.34
In vitro fertilization (IVF)	90 (7.4)	78 (6.2)	0.23
Alcohol during pregnancy	83 (6.8)	75 (5.9)	0.38
Smoke during pregnancy	74 (6.1)	88 (7.0)	0.38
Maternal COVID-19 infection in pregnancy	0 (0.0)	50 (3.9)	**<0.001**

However, we found a statistically significant difference in the number of antenatal check-ups [8.2 (1.3) vs 7.9(1.5), p<0.001] and ultrasound scans [7.7 (2.0) vs 7.3 (1.9), p<0.001] and in gestational age at the time of the first antenatal check-up [6.7 (1.6) vs 6.9 (1.3), p=0.002], as shown in Table 1.

There were no differences in pregnancy complications. Differences emerged in labor initiation, with a higher rate of labor induction during the COVID-19 pandemic [378 (31.1) vs 436 (34.6), p=0.008], although we found no differences in delivery mode. The number of health workers per patient during childbirth was not significantly reduced during 2020 [3.7 (1.3) vs 3.6 (1.4), p=0.03], except for the number of nurses [1173 (96.5) vs 1189 (94.3), p=0.009]. The number of women whose partner/relative did not enter the delivery room was also higher in 2020 [50 (4.1) vs 140 (11.1), p<0.001].

There were no differences in maternal or neonatal outcomes, as shown in Tables 2 and 3, however during lockdown there was a higher rate of mother and newborn skin-to-skin contact [897 (73.8) vs 1036 (82.2), p<0.001] and of breastfeeding within 2 hours from birth [830 (68.3) vs 1003 (79.6), p<0.001]

**Table 2:** Delivery characteristics during pandemic lockdown period compared with the same period in 2019

*Characteristics*	*2019 (n=1215) n (%)*	*2020 (n=1260) n (%)*	*p*
**Delivery at home**	1 (0.08)	1 (0.08)	0.98
**Labor mode**			**0.008**
Spontaneous	540 (44.4)	483 (38.3)	
Induced	378 (31.1)	436 (34.6)	
No labor	297 (24.5)	341 (27.1)	
**Delivery**			
Vaginal	656 (53.9)	715 (56.7)	0.07
Emergency CS	162 (13.3)	133 (10.5)	
Elective CS	315 (25.9)	345 (27.4)	
Ventouse	82 (6.7)	67 (5.3)	
**Health workers in the delivery ward,** mean ± SD	3.7 ± 1.3	3.6 ± 1.4	0.34
Midwife	1214 (99.9)	1259 (99.9)	0.23
Obstetric	818 (67.3)	804 (63.8)	0.07
Pediatrician	664 (54.7)	653 (51.8)	0.15
Anesthetist	643 (52.9)	627 (49.8)	0.12
Nurse	1173 (96.5)	1189 (94.3)	**0.009**
No partner/relatives at time of delivery	50 (4.1)	140 (11.1)	**<0.001**

CS: caesarian section.

**Table 3:** Maternal and neonatal outcome during pandemic lockdown period compared with the same period in 2019

*Outcomes*	*2019 (n=1215) n (%)*	*2020 (n=1260) n (%)*	*p*
**Maternal outcome**			
Epidural	607 (49.9)	622 (49.3)	0.77
Episiotomy	322 (26.5)	352 (27.9)	0.42
3rd and 4th perineal tears	4 (0.3)	6 (0.05)	0.55
Skin-to-skin	897 (73.8)	1036 (82.2)	<0.001
Breastfeeding within 2 h	830 (68.3)	1003 (79.6)	<0.001
Postpartum hysterectomy	1 (0.1)	1 (0.1)	0.98
Shoulder dystocia	3 (0.2)	0 (0.0)	0.78
Haematic loss >1500 cc	9 (0.7)	14 (1.1)	0.34
Return to operating room	11 (0.9)	3 (0.2)	0.03
**Neonatal outcome**			
Gestational age at birth (weeks)	38.7 (1.6)	38.5 (1.8)	0.15
Gestational age at birth (weeks)			0.36
≥28 and <34	15 (1.2)	21 (1.7)	
<28	2 (0.2)	5 (0.4)	
Neonatal birth weight (g), mean ± SD	3247.7 ± 483.9	3224.4 ± 514.1	0.25
Neonatal resuscitation	35 (2.8)	22 (2.5)	0.60
Admission to NICU	6 (0.5)	4 (0.3)	0.59
APGAR at 5 min <7	5 (0.4)	5 (0.4)	0.95
Stillbirth	0 (0.0)	3 (0.2)	0.09
Arterial PH <7	5 (0.4)	4 (0.3)	0.70
Unknown SGA	5 (0.4)	17 (0.2)	0.24

No cases of uterine rupture, eclampsia, maternal thromboembolism or stillbirth were reported during the lockdown period (Table 2).

## DISCUSSION

In our experience, the creation of two separate areas for infected and non-infected patients within the delivery ward enabled us to guarantee a safe assistance to birth to a high number of patients, without negatively influencing maternal and neonatal outcomes.

The greater gestational age at the time of the first antenatal check-up during 2020 is probably due to a reduction in availability of hospital staff, which was relocated to COVID-19 departments.

The lower number of obstetric check-ups and ultrasound scans performed in pregnancy during lockdown is probably due to women’s fear of contracting the infection in hospitals. The alarming effect that arose from the media reporting the struggle of the National Health Service and constantly displaying dramatic images of hospitalized patients in intensive care units critically increased the fear of the population. A reduction in the number of medical check-ups during lockdown has been reported in many medical fields, including cardiology^13,14^.

We assumed that the higher rate of induction of labor was a consequence of the need to organize the work of the delivery room, allowing a homogeneous redistribution of workload during shifts and, above all, allowing spatial and temporal separation of infected patients from non-infected patients.

The lower number of nurses in the delivery ward was due to the relocation of health workers to COVID-19 intensive care units. However, we managed to maintain an adequate number of obstetricians, gynecologists and anesthetists per patient.

The lower number of partners/relatives assisting birth in the delivery room is a consequence of the questionnaire screening we conducted at time of admission, as those who resulted positive were not allowed to enter the maternity ward. The enhancement of skin-to-skin contact and breastfeeding within 2 hours from birth is probably linked to the presence of a reduced number of relatives during the postpartum period.

The main strength of this study is that it was conducted on a large number of patients and in one of the six reference centers for the treatment of COVID-19 in women in Lombardy, the first region to be affected by the disease in Europe and one of the areas with the highest diffusion rate of infection.

## CONCLUSIONS

Despite the Sars-CoV-2 pandemic, with delivery room reorganization and relocation of resources, we were able to provide the same management as the previous year, guaranteeing an average of 20 births per day, with similar maternal and neonatal outcomes.

## CONFLICTS OF INTEREST

The authors have completed and submitted the ICMJE Form for Disclosure of Potential Conflicts of Interest and none was reported.

## FUNDING

There was no source of funding for this research.

## ETHICAL APPROVAL AND INFORMED CONSENT

The study was approved by the Institutional Review Board of Fondazione IRCCS Ca’ Granda, Ospedale Maggiore Policlinico, Milan, Italy (No.1512; date: April 2020). All participants provided written informed consent.

## DATA AVAILABILITY

The data supporting this research is available from the authors on reasonable request.

## AUTHORS’ CONTRIBUTIONS

All authors contributed significantly to the conception, planning, carrying out and analysis of the study. NC and FD were the primary writers of the manuscript. All authors read, revised and agreed to the publication of the final manuscript.

## PROVENANCE AND PEER REVIEW

Not commissioned; externally peer reviewed.
